# Lateral Size Governs Hydrogen Evolution Reaction Mechanisms and Kinetics of Ultrathin Pd Nanosheets in Acidic and Alkaline Electrolytes

**DOI:** 10.1021/acs.jpcc.6c03229

**Published:** 2026-07-27

**Authors:** Huiyu Gai, Hengrui Ma, Christian M. Schott, Elena L. Gubanova, Haiting Yu, Xiaomeng Xu, Eva Kolibalova, Moritz Gruber, Peter M. Schneider, Jian Zhou, Jan M. Macak, Zhaoxiong Xie, Aliaksandr S. Bandarenka

**Affiliations:** † Physics of Energy Conversion and Storage, 84665 Technical University of Munich, James-Franck-Str. 1, Garching 85748, Germany; ‡ College of Chemistry and Chemical Engineering, 12466 Xiamen University, Siming South Rd. 422, Xiamen 361005, China; § Central European Institute of Technology, 48252 Brno University of Technology, Purkyňova 123, Brno 61200, Czech Republic; ∥ Center of Materials and Nanotechnologies, University of Pardubice, Nam. Cs. Legii 565, Pardubice 530 02, Czech Republic; ⊥ Catalysis Research Center, Technical University of Munich, Ernst -Otto-Fischer-Straße 1, Garching 85748, Germany

## Abstract

Understanding the influence of nanoscale geometry on hydrogen evolution reaction (HER) mechanisms and kinetics is crucial for rational catalyst design. Here, we construct a well-defined model system of ultrathin Pd nanosheets with controlled lateral sizes (ca. 11 to 44 nm) and comparable thickness to systematically probe lateral size effects on HER kinetics in different electrolytes. Electrochemical impedance spectroscopy (EIS) was employed to analyze size-dependent interfacial kinetics and reaction mechanisms. In an acidic electrolyte, HER proceeds through a Volmer–Heyrovsky-dominated mechanism with a parallel Volmer–Tafel pathway, where lateral size influences both the intrinsic kinetics and the relative kinetic contribution. In alkaline electrolytes, HER kinetics are dominated by adsorption-related interfacial processes, and the lateral size affects the interfacial adsorption resistance. These results demonstrate that lateral size, a key geometric parameter, regulates HER mechanisms and kinetics under both acidic and alkaline conditions.

## Graphical abstract

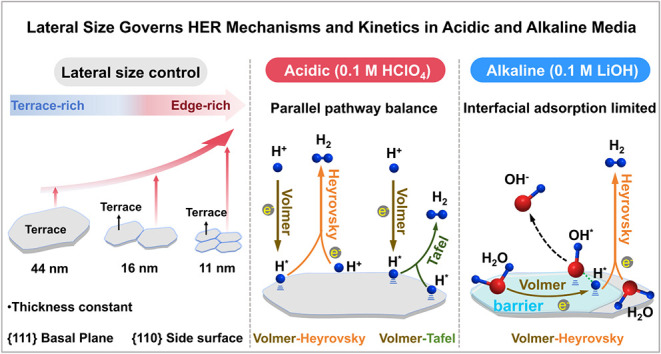

## 1 Introduction

The hydrogen evolution reaction (HER) is a cornerstone of sustainable hydrogen production via electrochemical water splitting technologies, offering a viable alternative to conventional fossil fuels. However, HER kinetics are highly sensitive to the nanoparticle shape, size, and electrolyte environment, leading to distinct reaction behaviors under acidic and alkaline conditions.^1,2^ At the mechanistic level, HER in both acidic and alkaline electrolytes is commonly described by two fundamental reaction pathways, namely the Volmer–Heyrovsky and Volmer–Tafel mechanisms. In acidic electrolytes, the Volmer step corresponds to electrochemical proton adsorption (H^+^ + e^–^ → H*), followed by either electrochemical desorption via the Heyrovsky step (H* + H^+^ + e^–^ → H_2_) or chemical recombination through the Tafel step (H* + H* → H_2_).^3^ In alkaline electrolytes, the Volmer step involves water dissociation (H_2_O + e^–^ → H* + OH*, OH* → OH^–^), while hydrogen evolution proceeds through either the Heyrovsky reaction with water as the proton donor (H* + H_2_O + e^–^ → H_2_ + OH^–^) or the Tafel recombination of adsorbed hydrogen species.^4^ This water dissociation step may introduce an additional energy barrier, resulting in sluggish kinetics.^5,6^ Therefore, understanding how catalyst structure influences HER reaction mechanisms and associated kinetic barriers in both acidic and alkaline electrolytes is central to the rational design of efficient HER electrocatalysts.

Palladium (Pd) has been widely used as an ideal model system for mechanistic studies of HER, owing to its Pt-like electronic structure and pronounced sensitivity of catalytic behavior to surface structure.^7–11^ Extensive efforts have been devoted to improving the HER performance of Pd-based catalysts by modulating both intrinsic and extrinsic properties, such as facet engineering,^12–14^ size control,^15–17^ and alloying.^18,19^ Among these approaches, reducing particle size to the nanoscale or even the atomic level is often associated with enhancing HER performance. This improvement is commonly attributed to an increased fraction of exposed, accessible surface area and to an enrichment of low-coordinated metal sites, such as edges and corners, which influence hydrogen adsorption and desorption kinetics.^15,20^ However, in conventional Pd nanoparticles, variations in particle size are accompanied by simultaneous changes in electrochemical surface area, facet exposure, and overall morphology. This strong coupling between multiple structural parameters makes it difficult to isolate the intrinsic role of lateral size in governing hydrogen interaction behavior and HER kinetics. In contrast, ultrathin two-dimensional (2D) Pd nanosheets provide a model system in which thickness and crystallographic facets remain essentially unchanged, whereas lateral size can be independently tuned.^21,22^ As a result, lateral size serves as a distinct geometric descriptor for correlating nanosheet structure with HER-related interfacial behavior and kinetics.

Herein, we construct a well-defined model catalyst platform based on ultrathin Pd nanosheets. Structural characterization reveals a comparable thickness of approximately 2 nm and preserved crystallographic facets, with only the lateral size precisely varied from 44 to 11 nm. This structural uniformity allows lateral size to serve as an independent geometric descriptor, independent of thickness, surface area, and facet contributions. Building on this model system, we employ an evaluation of electrochemical performance and electrochemical impedance spectroscopy to systematically investigate how lateral size regulates HER kinetics in both acidic and alkaline media. Our study demonstrates that HER follows the same fundamental mechanism across different lateral sizes, while lateral size influences different kinetic processes in acidic and alkaline electrolytes. In acidic media, the lateral size of nanosheets primarily governs intrinsic reaction kinetics and the relative contributions of parallel reaction pathways, whereas in alkaline electrolytes, interfacial adsorption processes are strongly dependent on lateral size. These results highlight lateral size as an important structural parameter for tuning HER kinetics on ultrathin Pd nanosheets and provide useful guidance for the design of 2D Pd electrocatalysts applicable to both acidic and alkaline conditions.

## 2 Experimental Section

### 2.1 Chemicals

Palladium (II) acetylacetonate (Pd­(acac)_2_, 99%), polyvinylpyrrolidone (PVP, K30), hexadecyltrimethylammonium bromide (CTAB, ≥98%), tungsten hexacarbonyl (W­(CO)_6_, 97%), and citric acid (CA, ≥99.5%) were purchased from Sigma-Aldrich. N,N-Dimethylformamide (DMF, ≥99.98%) was obtained from Fisher Chemical.

### 2.2 Synthesis of Pd NSs with Different Sizes

Pd nanosheets (Pd NSs) with different lateral sizes were synthesized based on previously reported methods with minor modifications.^23^ In a typical synthesis of the Pd NSs_44 sample, Pd­(acac)_2_ (10 mg, 32.8 μmol), PVP (30 mg), CTAB (60 mg, 164.6 μmol), and CA (10 mg, 47.6 μmol) were added to 10 mL of DMF in a glass vial. The mixture was magnetically stirred at room temperature for 1 h to form a homogeneous solution. Subsequently, W­(CO)_6_ (100 mg, 284.2 μmol) was introduced under argon atmosphere, and the reaction mixture was heated to 80 °C and maintained at this temperature for 1 h. After naturally cooling to room temperature, the resulting product was collected by centrifugation and washed with acetone, deionized water, and ethanol.

Pd NSs_16 and Pd NSs_11 were synthesized under identical conditions, except that the amount of CA was increased to 90 mg (428.3 μmol) and 170 mg (809.1 μmol), respectively, to regulate the nanosheet size.

### 2.3 Preparation of Pd NSs@C with Different Sizes

Vulcan XC72-R carbon (Cabot, USA) was employed as the support material. Prior to Pd loading, the Vulcan carbon was pretreated to enhance its surface hydrophilicity.^24^ Specifically, the carbon powder was immersed in a 30 wt % H_2_O_2_ solution and heated at 60 °C under a continuous stirring for 6 h. After treatment, the carbon was collected by the vacuum filtration and thoroughly washed with deionized water several times to remove residual oxidants. The pretreated carbon was then dried in an oven at 60 °C for further use.

Pd NSs@C catalysts were prepared by depositing the as-synthesized Pd nanosheets onto the pretreated carbon support with a Pd loading of ca. 20 wt %. In a typical procedure, an appropriate amount of pretreated Vulcan XC72-R carbon was dispersed in ethanol and ultrasonicated in an ice bath (below 40 °C) for 1 h to obtain a uniform suspension. Subsequently, a concentrated dispersion of Pd NSs was added dropwise to the above suspension, followed by further ultrasonication in an ice bath for another 1 h to ensure homogeneous deposition of Pd nanosheets onto the carbon support. The resulting product was collected by centrifugation, washed with ethanol several times, and finally dried at 80 °C to obtain the Pd NSs_11@C, Pd NSs_16@C, and Pd NSs_44@C catalysts.

### 2.4 Thermogravimetric Analysis

Thermogravimetric analysis (TGA) was performed on a Mettler Toledo thermogravimetric analyzer to determine the Pd mass loading of the Pd NSs@C catalysts. The sample weight was monitored as a function of temperature under a programmed heating protocol consisting of three sequential steps. Initially, residual moisture was removed by holding the sample at 135 °C for 15 min under an Ar atmosphere. Subsequently, the carbon support was completely oxidized at 800 °C in an O_2_ atmosphere for 30 min, during which metallic Pd was simultaneously converted to PdO. Finally, PdO was decomposed to metallic Pd (80%) by switching the atmosphere to Ar and maintaining the temperature at 800 °C for another 30 min. The heating rate and gas flow rate were fixed at 20 °C min^–1^ and 20 mL min^–1^, respectively.

Based on the TGA results, the Pd loadings were determined to be 19 wt % for Pd NSs_11@C, 22 wt % for Pd NSs_16@C, and 21 wt % for Pd NSs_44@C. These experimentally determined Pd loadings were used to normalize the HER current densities, enabling accurate calculation of the mass activity for each catalyst.

### 2.5 Electrochemical Measurements

All electrochemical experiments were conducted at ambient temperature using a conventional three-electrode configuration controlled by a VSP-300 potentiostat (Bio-Logic, France). Prior to use, the electrochemical cell was thoroughly cleaned to eliminate surface contaminants by sequential treatment with freshly prepared piranha solution (H_2_SO_4_/H_2_O_2_ = 2:1, v/v), followed by rinsing with hot deionized water, and then cold deionized water.

A glassy carbon disk electrode (5 mm diameter) served as the working electrode, while a mercury–mercury sulfate electrode (MMS, SI Analytics, Germany) and a spring-shaped Pd wire (Ø = 0.25 mm, 99.95%, MaTeck, Germany) were employed as the reference and counter electrodes, respectively. A Luggin capillary was used to minimize the ohmic drop between the reference and working electrodes. For experiments requiring hydrodynamic control, the working electrode was mounted onto an external rotation system (OrigaTrod, OrigaLys ElectroChem SAS, France).

Before catalyst deposition, the glassy carbon electrode was mechanically polished using alumina slurries with decreasing particle sizes (1.0, 0.3, and 0.05 μm), followed by rinsing with deionized water. Catalyst inks were prepared by dispersing 5 mg of the dried Pd/C catalyst in a mixed solvent consisting of 1800 μL deionized water, 723 μL isopropanol, and 15 μL Nafion solution (5 wt %). The mixture was ultrasonicated for 0.5 h to obtain a homogeneous suspension, and prior to each drop-casting step, the catalyst ink was briefly ultrasonicated for 3 min to ensure homogeneous dispersion. For electrochemical testing, 10 μL of the ink was deposited onto the polished glassy carbon surface (geometric area: 0.196 cm^2^) and dried under continuous rotation using a stream of warm air to ensure uniform film formation.

HER measurements were performed in H_2_-saturated 0.1 M HClO_4_ and 0.1 M LiOH electrolytes using a rotating disk electrode configuration. Prior to electrochemical measurements, the electrolyte and the three-electrode cell were purged with high purity Ar for at least 30 min while rotating the working electrode at 400 rpm to remove dissolved oxygen. The uncompensated solution resistance (R_u_) was subsequently determined by electrochemical impedance spectroscopy (EIS) under Ar atmosphere. The EIS measurements were carried out by introducing a shunt capacitor between the reference electrode and a Pt wire attached to the tip of the Luggin capillary, using a 25 mV sinusoidal perturbation over a frequency range of 100 kHz to 10 Hz. The obtained R_u_ values were used for *iR*_u_ correction of all HER performance data. Subsequently, the baseline electrochemical characterizations were performed by cyclic voltammetry (CV). The catalyst layer was electrochemically activated by repeated CV scans at a scan rate of 50 mV s^–1^ within a potential window covering the characteristic surface processes of Pd to establish a stable and reproducible surface state. The electrolyte was subsequently saturated with high-purity H_2_ for at least 20 min. HER activity was then evaluated by recording CVs at a scan rate of 10 mV s^–1^ under a rotation speed of 1600 rpm. The applied potential window ranged from −0.13 to 0.52 V vs RHE in acidic media and from −0.13 to 0.98 V vs RHE in alkaline media. Each experiment consisted of five successive scans, and the second scan was used for performance comparison. Different evaluation potentials were selected to ensure measurable and stable HER currents under acidic and alkaline conditions, namely −20 mV vs RHE in acidic media and −80 mV vs RHE in alkaline media.

Tafel plots were obtained by replotting the *iR*-corrected HER polarization curves as overpotential (η) versus *log*(*j*). The linear region in the kinetically controlled regime was fitted using the Tafel equation η = *a* + *b log*(*j*), from which the Tafel slope (*b*) and exchange current density (*j*_0_ = 10^–*a*/*b*^) were extracted. The reported *b* and *j*_0_ values represent the average of different measurements with error bars corresponding to the standard deviation.

Additional EIS measurements were carried out after HER measurement at selected potentials to probe the HER kinetics and mechanistic features. In these measurements, a perturbation amplitude of 10 mV was applied after holding the electrode at the target potential for 5 min to ensure equilibrium conditions and complete hydride formation in Pd. The investigated potential range was adjusted according to the electrolyte (0 to −20 mV vs RHE in acidic solution and 0 to −80 mV vs RHE in alkaline solution). The impedance spectra were analyzed using homemade software,^25^ and the validity of the fitted data was confirmed using Kramers–Kronig consistency tests, with the root-mean-square deviation (RMSD) values below 2%, confirming the reliability of the fitted results.

The electrochemically active surface area (ECSA) of the Pd NSs@C catalysts was determined by CO stripping voltammetry in 0.1 M HClO_4_. The electrolyte was first saturated with a CO/Ar gas mixture (1000 ppm of CO) for 15 min, followed by holding the potential at 0.1 V vs RHE for 1 h 7 min under rotation to allow CO adsorption. After purging dissolved CO with Ar, CO oxidation was recorded by cyclic voltammetry without electrode rotation. The ECSA was obtained by integrating the CO oxidation peak after background subtraction, assuming a charge density of 420 μC cm^–2^.^26^ The electrochemical specific surface area was calculated by normalizing the ECSA to the Pd mass on the electrode.

### 2.6 Surface Science Characterization

Transmission electron microscopy (TEM) was performed with JEOL JEM 2100, which was operated at 200 kV. The as-prepared Pd samples were dispersed in ethanol and dropped on Cu grid with ultrathin carbon on Lacey carbon support film. In addition, Pd NSs and Pd NSs@C were examined at the nanoscale using a Cs-corrected transmission electron microscope (TITAN Themis 60–300 Cubed, Thermo Fisher Scientific, USA). High-resolution imaging at subångström resolution was performed at 300 kV using a CETA camera. Corresponding Fast Fourier Transform (FFT) patterns were analyzed to determine crystallographic orientations and lattice features.

X-ray photoelectron spectroscopy (XPS) was used to analyze the surface chemical composition and electronic structure of the Pd NSs@C catalysts. The measurements were carried out on a SPECS system equipped with an XR 50 M X-ray source using a monochromatic Al Kα anode (*hν* = 1486.74 eV). Photoelectrons emitted from the sample surface were collected by a hemispherical energy analyzer (SPECS, Germany), allowing precise determination of their kinetic and corresponding binding energies. The binding energy scale was calibrated against the C 1s peak at 284.8 eV. High-resolution spectra were obtained for the Pd 3d, C 1s, and O 1s regions, and all spectra were processed by applying a Shirley background and subsequent peak fitting.

The crystalline structure and phase composition of the Pd NSs@C catalysts were examined by powder X-ray diffraction (XRD). The measurements were carried out using a Rigaku MiniFlex 600-C diffractometer equipped with a Cu Kα radiation source (λ = 1.5406 Å). Diffraction patterns were collected over a 2θ range of 30–90° using a continuous scan mode with a scanning rate of 10°/min.

## 3 Results and Discussion

### 3.1 Structural and Morphological Characterization of Size-Controlled Pd Nanosheets

The size-controlled Pd NSs were synthesized through a reported wet-chemical, CO-mediated synthesis method.^23^ Figure 1 presents representative TEM images of the Pd NSs, along with the corresponding lateral size distributions. All samples display well-defined 2D morphologies with the average lateral size decreasing systematically from ∼44 nm to ∼16 nm and ∼11 nm, hereafter denoted as Pd NSs_44, Pd NSs_16, and Pd NSs_11, respectively. As evidenced by TEM images, Pd NSs_44 exhibit extended and relatively flat basal terraces, whereas Pd NSs_16 and Pd NSs_11 show progressively increased edge irregularity. This evolution reflects a higher density of edge and low-coordinated surface sites in the smaller nanosheets, which is an intrinsic geometric consequence of lateral size reduction. Such changes are particularly relevant for hydrogen electrocatalysis, where adsorption-related processes are sensitive to local coordination environments.^27,28^

**Figure 1:**
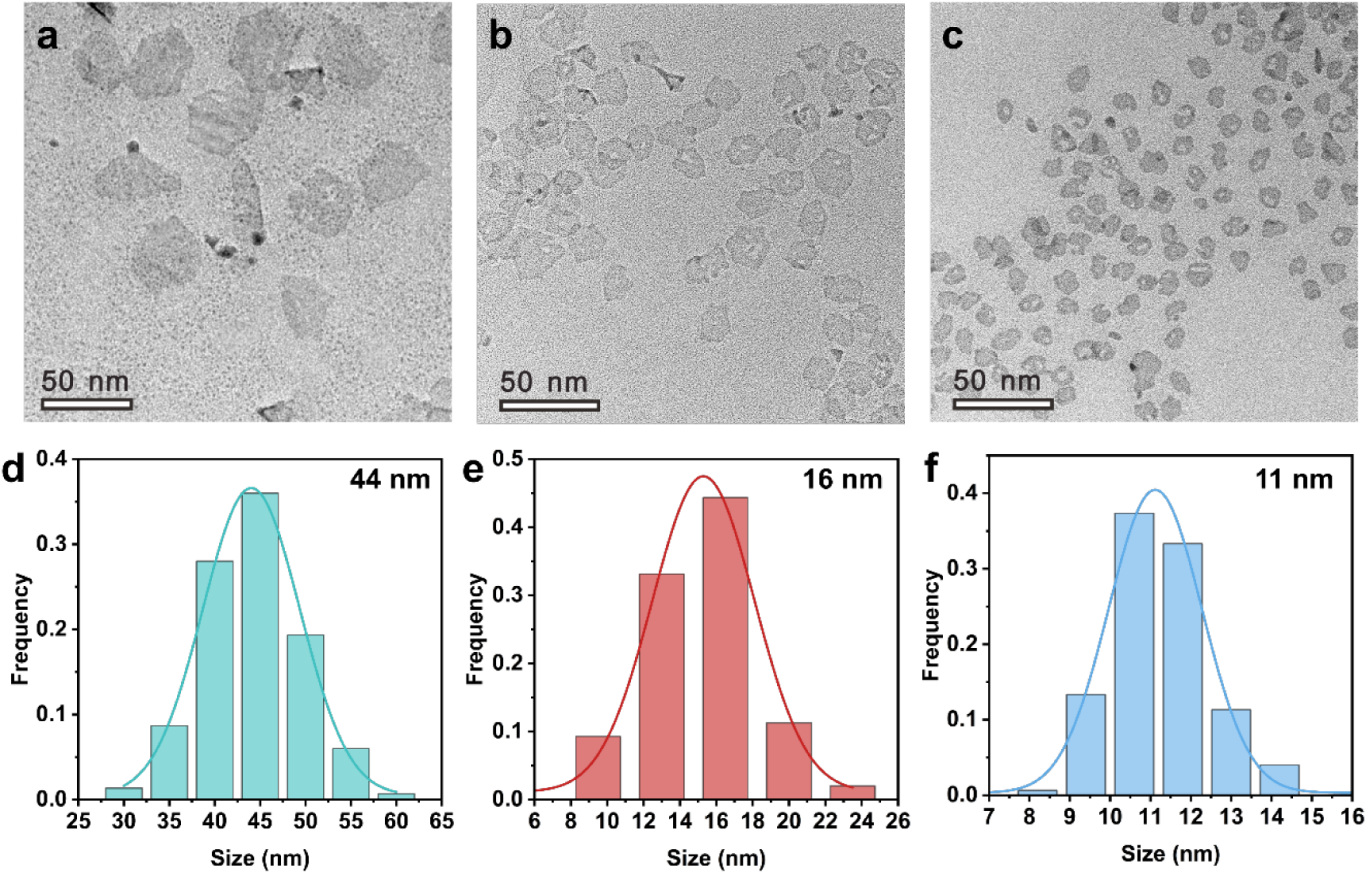
TEM images of (a) Pd NSs_44, (b) Pd NSs_16, and (c) Pd NSs_11 and (d–f) the corresponding histograms of the lateral size distributions, each constructed from statistical counting of 150 NSs.

To decouple lateral size effects from thickness-related contributions, the vertical dimensions of the Pd NSs were examined by side-view high-resolution TEM (HRTEM), as shown in Figure S1. All samples exhibit parallel 9–12 atomic layers with measured thicknesses of approximately 1.9–2.4 nm,^29^ confirming that they are genuinely ultrathin nanosheets rather than thick platelet-like particles with significant vertical dimensions. The comparable thickness across Pd NSs_44, Pd NSs_16, and Pd NSs_11 indicates that lateral size varies independently, which serves as a valid model for elucidating the size effect of Pd NSs in electrocatalytic reactions.

Detailed structural characteristics were further investigated using top-view HRTEM combined with corresponding FFT analysis (Figure 2a–b, e–f, i–j). In the extracted FFT patterns, characteristic diffraction spots, such as, (202), (220), (022̅) and (0̅2̅2) can be identified and indexed to [1̅11] zone axis, confirming that the basal surfaces of all samples are predominantly terminated by {111} facets. In addition, the unique diffraction spot of 1/3(422) was observed in the Pd NSs_44 sample, which indicates the existence of stacking faults due to the ultrathin structure.^30^ Side-view HRTEM and FFT patterns (Figure 2c–d, g–h, k–l) reveal lattice fringes and diffraction features that can be indexed to [011] zone axis, indicating that the sheet side surfaces primarily expose {110}-type facets. Taken together, this facet configuration, with {111} basal surfaces and {110}-type side surfaces, remains consistent across all size nanosheets, implying that the lateral size modulation primarily changes the density and coordination of edge sites rather than the facet identity.

**Figure 2:**
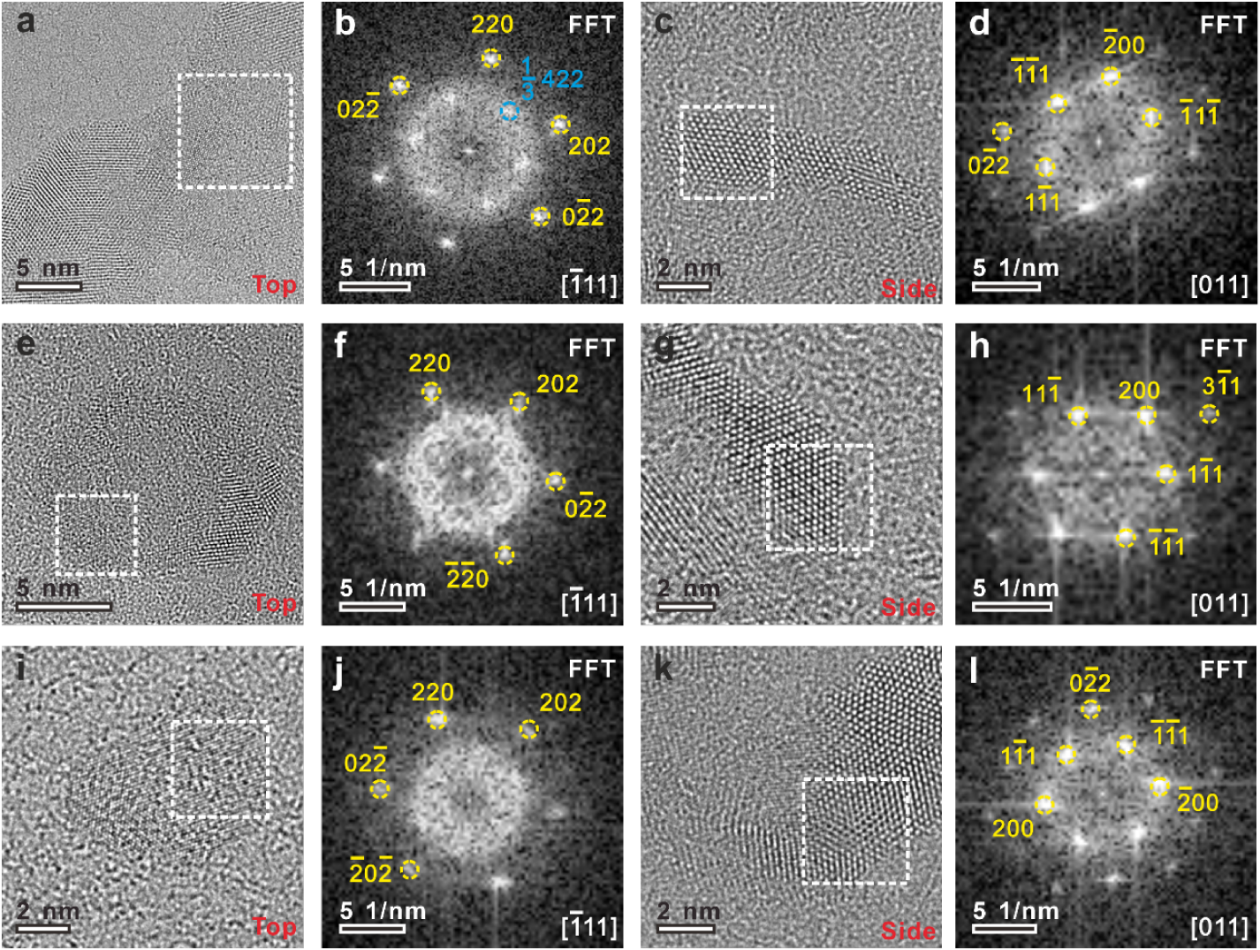
HRTEM images and corresponding FFT patterns of Pd NSs from top and side views. (a–d) Pd NSs_44. (e–h) Pd NSs_16. (i–l) Pd NSs_11. From left to right for each sample: top-view HRTEM, corresponding FFT pattern, side-view HRTEM, and corresponding FFT pattern.

These local crystallographic observations were further corroborated by XRD patterns of the carbon-supported Pd catalysts (denoted as Pd NSs@C) (Figure S2). In all cases, the diffraction features matched face-centered cubic (fcc) Pd (PDF#46-1043). No discernible diffraction peak shift was observed among Pd NSs_44@C, Pd NSs_16@C, and Pd NSs_11@C, indicating the absence of detectable average lattice expansion or contraction upon decreasing the lateral size.^31^ These results suggest that no measurable average lattice strain associated with lateral-size modulation in the pristine catalysts. XPS was used to probe the surface composition and oxidation states of Pd NSs@C catalysts. Survey spectra (Figure S3a) confirms Pd as the dominant element, together with signals from C and O originating from the carbon support and surface species. High-resolution Pd 3d spectra (Figure S3b–d) were deconvoluted into metallic Pd^0^ and oxidized Pd^2+^ components, with the Pd^2+^ doublets located at higher binding energies than the corresponding Pd^0^.^32^ A systematic increase in the Pd^2+^ fraction is observed as lateral size decreases, indicating enhanced surface oxidation of the smaller Pd NSs. As reported by Vogel et al., oxygen binds considerably more strongly to low-coordinated Pd defect sites than to terrace atoms, which rendering edge-rich Pd surfaces more susceptible to surface oxidation.^33^ The increased Pd^2+^ fraction is generally consistent with the increased proportion of low-coordinated edge sites in the smaller nanosheets.

Collectively, TEM, HRTEM/FFT, XRD, and XPS analyses establish a well-defined structural platform in which lateral size is the main tunable parameter, while thickness and crystallographic features are preserved. The combined structural and surface characterization consistently indicates that decreasing the lateral size primarily increases the proportion of low-coordinated edge sites rather than introducing measurable average lattice strain in the pristine catalysts. Such well-controlled structural and electronic characteristics enable a direct correlation between the lateral size and the electrochemical behavior in both acidic and alkaline media.

### 3.2 Electrochemical Surface Characteristics and Hydrogen-Related Features

Electrochemical surface characteristics and hydrogen-related features of the Pd NSs@C catalysts were investigated by CV, while the ECSA was determined through CO stripping voltammetry. These measurements provide insight into the electrochemically accessible Pd sites, hydrogen adsorption and desorption behavior, and electrolyte-dependent interfacial processes.

As summarized in Figure S4, all Pd NSs@C catalysts exhibit comparable specific surface areas (SSA), defined as the ECSA normalized to the Pd mass loading, indicating similar densities of electrochemically accessible Pd sites. Pd NSs_16@C shows a slightly higher SSA (38.4 ± 1.4 m^2^ g^–1^_Pd_) compared to Pd NSs_11@C (37.6 ± 0.5 m^2^ g^–1^_Pd_) and Pd NSs_44@C (35.3 ± 2.5 m^2^ g^–1^_Pd_). Figure 3a shows the ECSA-normalized CV profiles of Pd NSs_44@C, Pd NSs_16@C, and Pd NSs_11@C, recorded in Ar-saturated 0.1 M HClO_4_. All catalysts exhibit the characteristic electrochemical signatures of Pd, including a pronounced hydrogen adsorption/desorption (H_upd_) region at low potentials and Pd oxidation/reduction processes at higher potentials.^34^ Despite similar Pd site densities, noticeable size-dependent differences are observed in the H_upd_ region among the three nanosheet sizes; both the peak shapes and their relative intensities vary with the lateral size of the Pd NSs. This observation indicates that lateral size influences the hydrogen-related electrochemical behavior of the Pd nanosheets in acidic electrolyte. In 0.1 M LiOH, the CV profiles (Figure 3b) differ notably from those in acid due to the requirement of extra water dissociation.^35^ As a result, hydrogen adsorption and desorption features are significantly suppressed and shifted to more positive potentials, compared with those observed in the acidic electrolyte. In particular, the significantly weaker hydrogen-related response of Pd NSs_16@C suggests modified hydrogen adsorption and desorption behavior at individual Pd sites under alkaline conditions.

**Figure 3:**
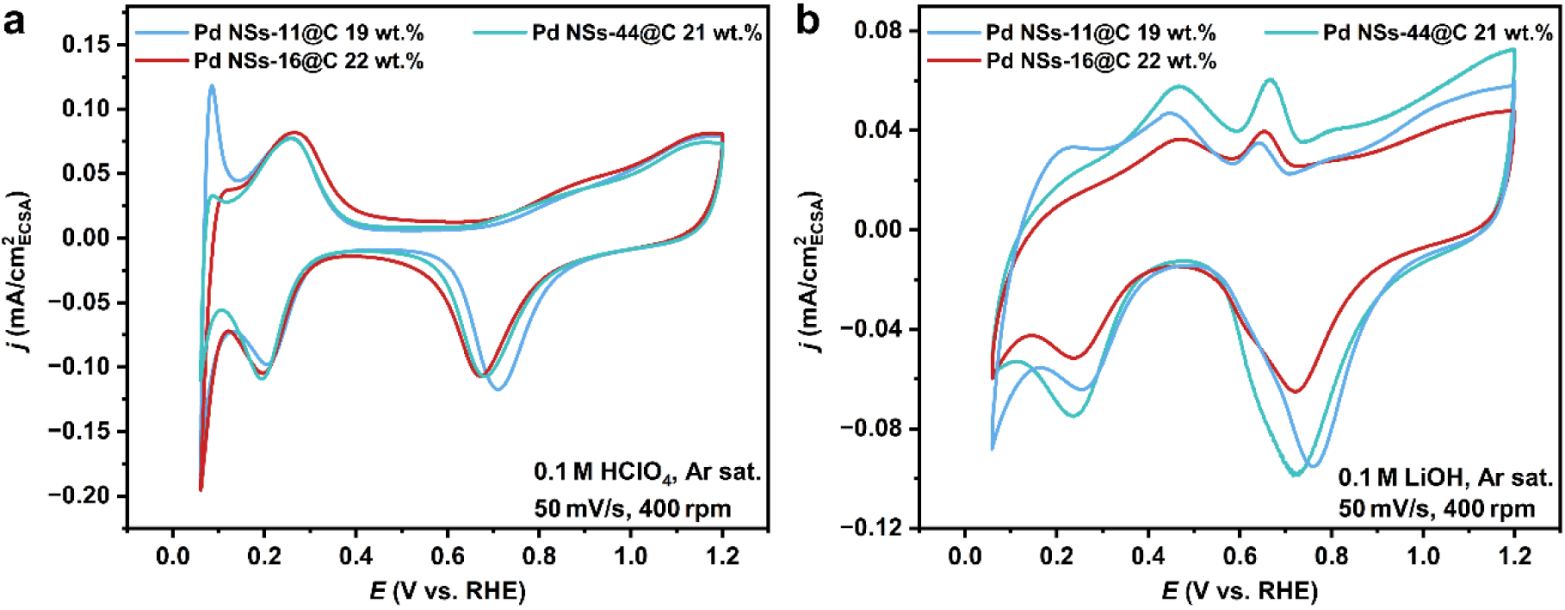
ECSA-normalized CVs of Pd NSs@C catalysts recorded in Ar-saturated (a) 0.1 M HClO_4_, (b) 0.1 M LiOH at a scan rate of 50 mV s^–1^ and a rotation speed of 400 rpm.

### 3.3 Hydrogen Evolution Reaction Performance in Acidic Electrolytes

The HER activity of the Pd NSs was evaluated in H_2_-saturated 0.1 M HClO_4_ using a rotating disk electrode at 1600 rpm. The *iR*-corrected polarization curves (Figure 4a) show a clear size dependence of acidic HER activity, following the orders of Pd NSs_11@C > Pd NSs_16@C > Pd NSs_44@C. This trend is further quantified by the mass activity (MA) and the specific activity (SA) at −20 mV vs RHE (Figure 4b and Table S1). Pd NSs_11@C exhibits the highest MA (51.8 ± 3.29 mA/mg_Pd_) and SA (137.5 ± 8.73 μA/cm^2^_ECSA_), compared with 27.9 ± 0.46 mA/mg_Pd_, 72.1 ± 1.19 μA/cm^2^_ECSA_ for Pd NSs_16@C and 22.6 ± 2.37 mA/mg_Pd_, 64.0 ± 6.73 μA/cm^2^_ECSA_ for Pd NSs_44@C. Despite possessing the largest SSA, as determined by CO stripping, Pd NSs_16@C shows no corresponding enhancement in either MA or SA, confirming that the size-dependent acidic HER performance is not easily governed by the number of electrochemically accessible Pd sites, but instead reflects intrinsic kinetic differences associated with lateral-size modulation.

**Figure 4:**
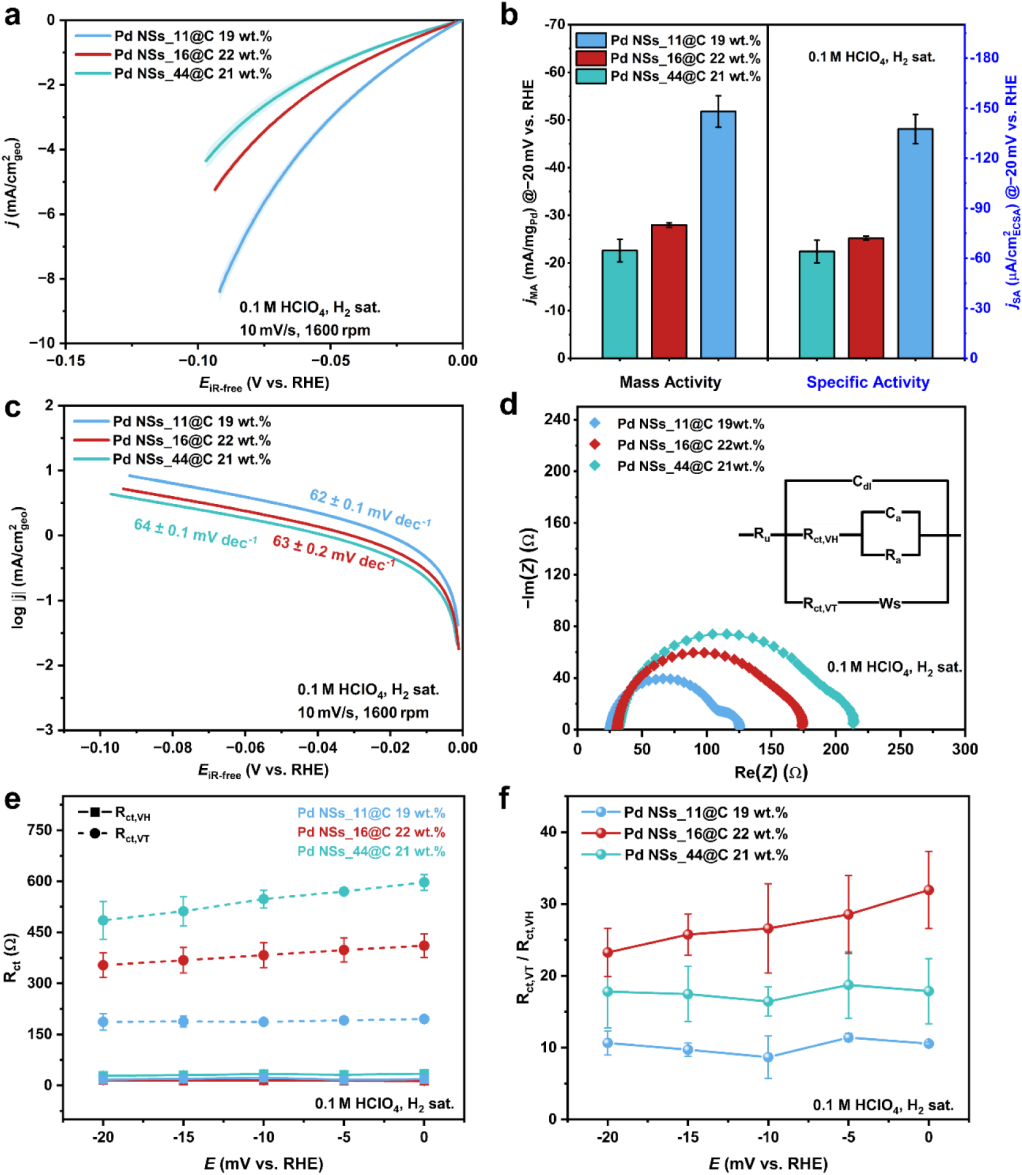
(a) HER polarization curves of Pd NSs@C catalysts in H_2_ saturated 0.1 M HClO_4_. (b) Mass and specific activity evaluated at −20 mV vs RHE. (c) Tafel plots. (d) Nyquist plots recorded at −20 mV vs RHE with corresponding EEC fits. (e) Charge transfer resistances (R_ct,VH_ and R_ct,VT_) derived from EIS, measured from 0 to −20 mV vs RHE. (f) R_ct,VT_ /R_ct,VH_ ratio illustrating the kinetic balance between the two parallel HER pathways.

The kinetic origin of this size dependence was elucidated by Tafel analysis (Figure 4c). In general, ideal Tafel slopes of approximately 30, 40, and 120 mV dec^–1^ are commonly assigned to the Tafel, Heyrovsky, and Volmer steps as the possible rate-determining steps (RDS) of the HER. However, under practical experimental conditions, the measured Tafel slopes typically deviate from these ideal values and may vary with the applied potential and nonideal surface coverage.^34,36^ In this context, all Pd NSs@C catalysts exhibit similar Tafel slopes of ca. 62 to 64 mV dec^–1^, suggesting a common dominant mechanism and the same RDS under acidic conditions. The geometric-normalized exchange current densities (*j*_0_), extracted from the Tafel plots show a clear size dependence (Figure S5a), with Pd NSs_11@C exhibiting the highest *j*_0_, followed by Pd NSs_16@C and Pd NSs_44@C. The higher *j*_0_ of smaller Pd NSs indicates more favorable HER kinetics and a reduced kinetic barrier. The same trend persists after normalization by ECSA (Figure S5b), confirming that the observed activity differences originate from intrinsic kinetic variations rather than surface area effects.

To further elucidate the origin of the size-dependent HER kinetics under acidic conditions, EIS measurements were performed from 0 to −20 mV vs RHE in H_2_-saturated 0.1 M HClO_4_. The complete potential-dependent Nyquist EIS plots are provided in Figure S6. Figure 4d displays the Nyquist plots of the three samples at −20 mV vs RHE together with the corresponding fits. The spectra were fitted using a physically meaningful equivalent electrical circuit (EEC) model (Figure S7) which has been successfully applied to investigate HER kinetics on polycrystalline Pt electrodes,^37^ Pd/C nanoparticles,^5^ and other metallic HER electrocatalysts.^38,39^ In this model, the uncompensated resistance, R_u_ accounts for the ohmic contributions from the electrolyte, while the nonfaradaic interfacial response is represented by a constant phase element (CPE), capturing the nonideal double-layer capacitive behavior commonly observed at the electrode–electrolyte interface. Notably, the model includes two parallel faradaic pathways corresponding to the Volmer–Heyrovsky (VH) and Volmer–Tafel (VT) of the HER. The VH pathway is described by a charge-transfer resistance (R_ct,VH_) in series with an adsorption-related branch composed of an adsorption resistance (R_a_) and an adsorption capacitance (C_a_) in parallel, which together reflect the kinetics and dynamic response of adsorbed species at the electrode surface. The VT pathway is characterized by a charge transfer resistance (R_ct,VT_) and a Warburg impedance (W_s_).^37^

As illustrated by the EEC model in Figure 4d, acidic HER is described by two parallel faradaic pathways, namely the VH and VT pathways. The fitted charge-transfer resistances are summarized in Figure 4e and Table S2. The R_ct,VH_ exhibits comparatively small variations (ca. 15–29 Ω) across the three Pd nanosheets, whereas the R_ct,VT_ exhibits a substantially larger (ca. 187–485 Ω) and size-dependence (Pd NSs_44@C > Pd NSs_16@C > Pd NSs_11@C). This difference is consistent with the distinct elementary processes involved in the two pathways. According to the established HER mechanism in acidic media, the Volmer step benefits from the abundance of solvated protons, enabling facile adsorption of hydrogen on the catalyst surface via direct proton-coupled electron transfer. Subsequently, hydrogen evolution proceeds through either the Heyrovsky step, where an adsorbed hydrogen intermediate undergoes electrochemical desorption to form H_2_, or the Tafel step, which requires the chemical recombination of two neighboring adsorbed hydrogen atoms prior to H_2_ desorption.^4^ Therefore, compared with the VH pathway, the VT pathway is intrinsically more sensitive to the local coordination environment. In the present well-controlled Pd nanosheet model system, the exposed facets, crystal structure, and nanosheet thickness remain essentially unchanged across the three Pd nanosheets. The primary structural variation introduced by decreasing lateral size is the increased proportion of low-coordinated edge sites, suggesting that the experimentally observed kinetic enhancement of the VT pathway is mainly associated with the higher density of low-coordinated edge sites. This is consistent with a recent atomistic study on Pd nanoparticles,^40^ which demonstrated that edge and corner sites facilitate H–H recombination and subsequent H_2_ desorption. These findings provide a mechanistic explanation for the experimentally observed decrease in R_ct,VT_ with decreasing lateral size.

To more directly evaluate the relative contributions of the two parallel pathways to the overall HER, the EIS-derived charge-transfer resistances were compared in terms of their ratio (R_ct,VT_ /R_ct,VH_).^37^ As shown in Figure 4f and summarized in Table S2, the R_ct,VT_ /R_ct,VH_ ratio differs considerably among the three Pd nanosheets, indicating the lateral-size modulation changes the kinetic balance between the parallel VH and VT pathways, thereby altering their relative contributions to the overall HER. Pd NSs_11@C exhibits the lowest R_ct,VT_ /R_ct,VH_ ratio, which indicates the largest relative contribution of the VT pathway. The lowest R_ct,VT_ in the smallest nanosheets, combined with a similarly low R_ct,VH_, both parallel VH and VT pathways are kinetically favorable. As the lateral size increases to Pd NSs_16@C, the pronounced increase in R_ct,VT_ markedly reduces the kinetic contribution of the VT pathway, whereas the R_ct,VH_ remains comparable to that of Pd NSs_11@C. HER kinetics become increasingly dominated by the VH pathway, with only a limited kinetic contribution from the VT pathway. Upon further increasing the lateral size to Pd NSs_44@C, the ratio decreases relative to Pd NSs_16@C, suggesting a moderate recovery of the relative kinetic contribution of the VT pathway. However, in the largest Pd NSs, both the VH and VT pathways exhibit the highest charge-transfer resistances, leading to the slowest HER kinetics. Therefore, lateral-size modulation does not alter the dominant role of the VH pathway but progressively changes the relative kinetic contribution of the VT pathway, giving rise to distinct kinetic characteristics among the three Pd nanosheets.

### 3.4 Hydrogen Evolution Reaction Performance in Alkaline Electrolyte

In contrast to acidic electrolytes, alkaline HER exhibits reduced activity and a different size dependence, reflecting a distinct kinetic regime under alkaline conditions. As shown in Figure 5a, HER activity follows the order of Pd NSs_11@C > Pd NSs_44@C > Pd NSs_16@C. Quantitative comparison at −80 mV vs RHE confirms the same trend in both mass- and ECSA-normalized activities (Figure 5b, Table S3). The reversal in the relative performance of Pd NSs_16@C and Pd NSs_44@C compared with acidic conditions highlights a pronounced electrolyte-dependent modulation of the size-activity relationship.

**Figure 5:**
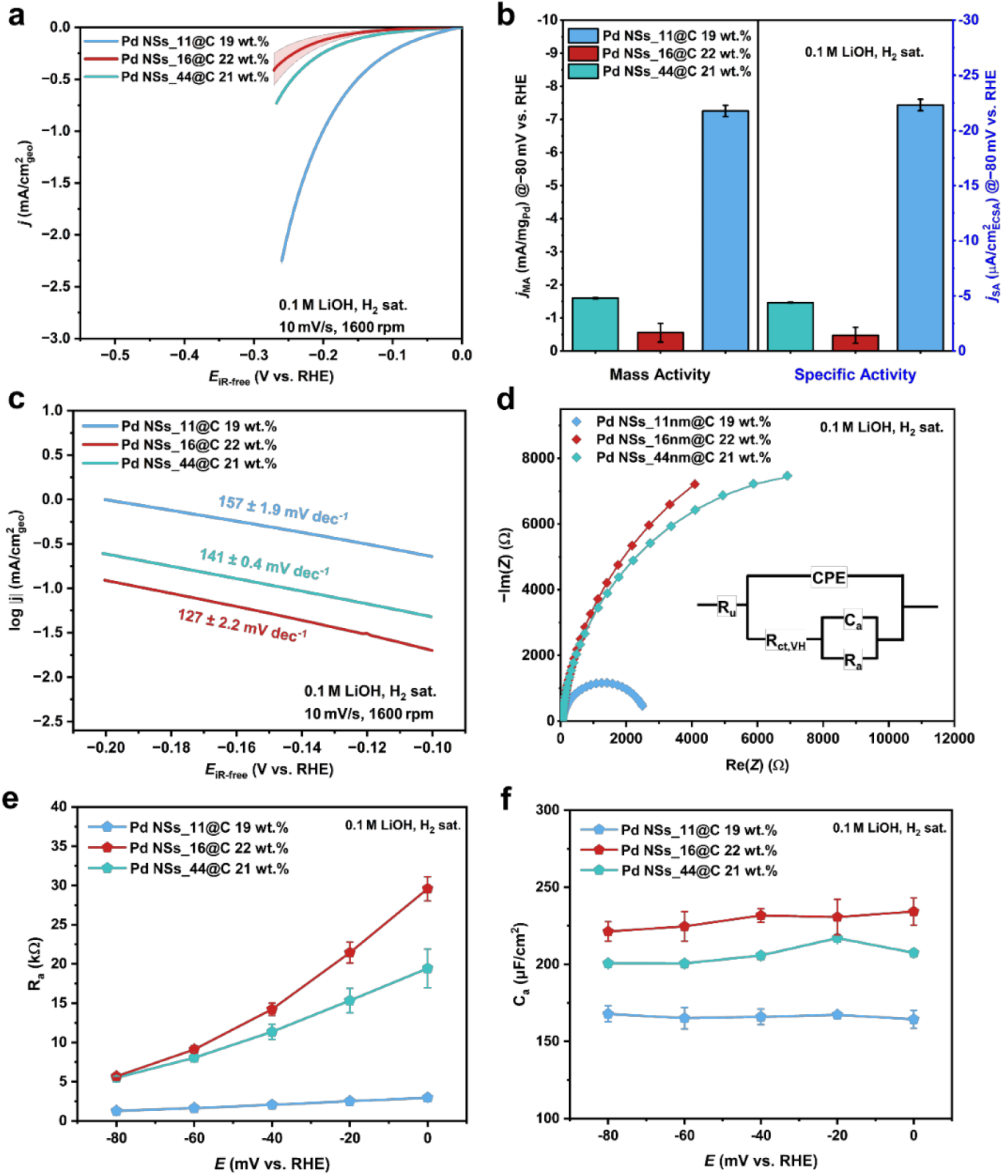
(a) HER polarization curves in H_2_ saturated 0.1 M LiOH. (b) Mass and specific activity evaluated at −80 mV vs RHE. (c) Tafel plots. (d) Nyquist plots with the fitted EEC model at −80 mV vs RHE. Potential-dependent (e) R_a_ and (f) C_a_ from EIS fitting from 0 to −80 mV vs RHE.

As widely recognized, HER in alkaline media relies on the proton generation from water molecules rather than from solvated protons under acidic conditions, rendering the Volmer step intrinsically more complex. As shown in Figure 5c, all Pd NSs@C catalysts exhibit larger Tafel slopes (∼127–157 mV dec^–1^). These values exceed the ideal Volmer-limit value of 120 mV dec^–1^, indicating nonideal alkaline HER kinetics beyond a simple Volmer-limited mechanism.^34,36^ As a reliable kinetic descriptor, the *j*_0_ values further elucidate the intrinsic HER kinetics in alkaline. As shown in Figure S8, both geometric area- and ECSA-normalized *j*_0_ follow the same size-dependent trend as the HER activity, with Pd NSs_11@C exhibiting the highest *j*_0_, followed by Pd NSs_44@C and Pd NSs_16@C. The highest *j*_0_ of Pd NSs_11@C reflects more efficient alkaline HER kinetics, whereas the lowest *j*_0_ of Pd NSs_16@C points to a larger kinetic barrier under alkaline conditions.

EIS provides further insight into the possible origin of the size dependence in alkaline media. Measurements were performed over a range of potentials (0 to −80 mV vs RHE), and representative Nyquist plots at −80 mV are shown in Figure 5d, with potential-dependent fitting results summarized in Figure S9. Under alkaline conditions, water molecules rather than solvated protons serve as the proton donor, and the Volmer step requires water adsorption and dissociation to generate adsorbed hydrogen together with OH-containing intermediates.^41^ The more demanding generation of adsorbed hydrogen significantly suppresses the contribution of the VT pathway, which relies on the recombination of neighboring adsorbed hydrogen atoms, contributes negligibly within the investigated potential range. Accordingly, the alkaline HER is described by a single VH pathway represented by R_ct,VH_ together with an adsorption-related R_a_/C_a_ pair.

As shown in Figure S10 and Figure 5e, across all Pd NSs@C catalysts, R_a_ falls in the kΩ range, which is two to 3 orders of magnitude larger than R_ct,VH_, indicating that adsorption-related interfacial processes dominate the overall alkaline HER kinetics rather than charge transfer. Previous studies have shown that alkaline HER kinetics are strongly influenced by interfacial processes involving water dissociation and the formation of hydrogen- and hydroxyl-containing intermediates, which can modify hydrogen adsorption energetics and surface reaction dynamics.^6,42^ As the adsorption-related capacitance connected in parallel with R_a_ within the VH equivalent circuit, C_a_ was also extracted from the EIS fitting results and is summarized in Figure 5f. Among the three catalysts, Pd NSs_11@C exhibits substantially lower R_a_ than the other two samples over the entire investigated potential range, whereas Pd NSs_16@C and Pd NSs_44@C display comparable R_a_ values with similar potential-dependent trends. A similar potential-dependent trend is observed for C_a_. Since R_a_ and C_a_ jointly characterize the adsorption-related interfacial response in the VH equivalent circuit, their combined variation indicates distinct adsorption-related behavior among the three Pd nanosheets. Specifically, Pd NSs_11@C exhibits R_a_ values of only ca. 1.3 to 3.0 kΩ, while Pd NSs_16@C and Pd NSs_44@C exhibit considerably higher values ranging from ca. 5.6 to 29.6 kΩ depending on the applied potential, indicating a markedly facilitated adsorption-related interfacial kinetics in the smallest nanosheets. These results suggest that reducing the nanosheet lateral size may facilitate the adsorption-related processes governing alkaline HER. However, the specific atomistic roles of edge sites in water dissociation and the management of OH-containing intermediates require further theoretical and operando investigations.

## 4 Conclusion

In this work, a series of ultrathin Pd nanosheets with precisely controlled lateral sizes was synthesized as a model system to probe size-dependent HER mechanisms and kinetics under both acidic and alkaline conditions. Electrochemical impedance spectroscopy provides direct insight into the distinct HER reaction mechanisms in acidic and alkaline electrolytes and clarifies the role of lateral size in regulating HER kinetics. In acidic electrolyte, all Pd nanosheets follow a common Volmer–Heyrovsky-dominated HER mechanism with a parallel Volmer–Tafel pathway, whose relative contribution is strongly dependent on nanosheet lateral size. The smallest Pd nanosheets exhibit the highest acidic HER activity, benefiting from favorable intrinsic kinetics together with a stonger contribution from the Volmer–Tafel pathway. In alkaline electrolytes, the HER kinetics are governed by adsorption-related interfacial processes. Lateral size modulation primarily affects the adsorption-related resistance. The minimized resistance of Pd NSs_11@C accounts for their superior alkaline HER activity, whereas larger nanosheets suffer from severe kinetic limitations. Overall, these findings establish that lateral size is an effective parameter for regulating hydrogen evolution kinetics on ultrathin Pd nanosheets in both acidic and alkaline electrolytes, providing mechanistic insights into structure-kinetics relationships and practical design guidance for two-dimensional metal electrocatalysts operating across diverse electrolyte environments.

## Supplementary Material



## Notes

Additional HRTEM characterization, XRD patterns, XPS analyses and fitting results, ECSA and SSA measurements, complete Nyquist plots in acidic and alkaline electrolytes, equivalent-circuit models, EIS fitting parameters, and HER performance data (PDF)
