# Supplementary material for: Lateral Size Governs Hydrogen Evolution Reaction Mechanisms and Kinetics of Ultrathin Pd Nanosheets in Acidic and Alkaline Electrolytes
Source: J Phys Chem C Nanomater Interfaces. 2026 Jul 27;130(31):10874–84. doi: 10.1021/acs.jpcc.6c03229 (PMC13455097; doi:10.1021/acs.jpcc.6c03229)
Supplement: Supplementary file 1 [file jp6c03229_si_001.pdf]

Supporting Information

Lateral Size Governs Hydrogen Evolution Reaction Mechanisms and Kinetics of Ultrathin Pd Nanosheets in Acidic and Alkaline Electrolytes

Huiyu Gai [#, a], *Hengrui Ma* [#, b], *Christian M. Schott* [a], *Elena L. Gubanova* [a], *Haiting Yu* [a], *Xiaomeng Xu* [a], *Eva Kolibalova* [c], *Moritz Gruber* [a], *Peter M. Schneider* [a], *Jian Zhou* [a], *Jan M. Macak* [c, d], *Zhaoxiong Xie* [b], *Aliaksandr S. Bandarenka* [*, a, e]

[a] Physics of Energy Conversion and Storage, Technical University of Munich, James-Franck-Str. 1, 85748 Garching, Germany

[b] College of Chemistry and Chemical Engineering, Xiamen University, Siming South Rd. 422, 361005, Xiamen, China

[c] Central European Institute of Technology, Brno University of Technology, Purkyňova 123, 61200 Brno, Czech Republic

[d] Center of Materials and Nanotechnologies, University of Pardubice, Nam. Cs. Legii 565, 530 02 Pardubice, Czech Republic

[e] Catalysis Research Center, Technical University of Munich, Ernst-Otto-Fischer-Straße 1, 85748 Garching, Germany

*Corresponding Author E-mail: bandarenka@ph.tum.de

[#] These authors contributed equally to this work

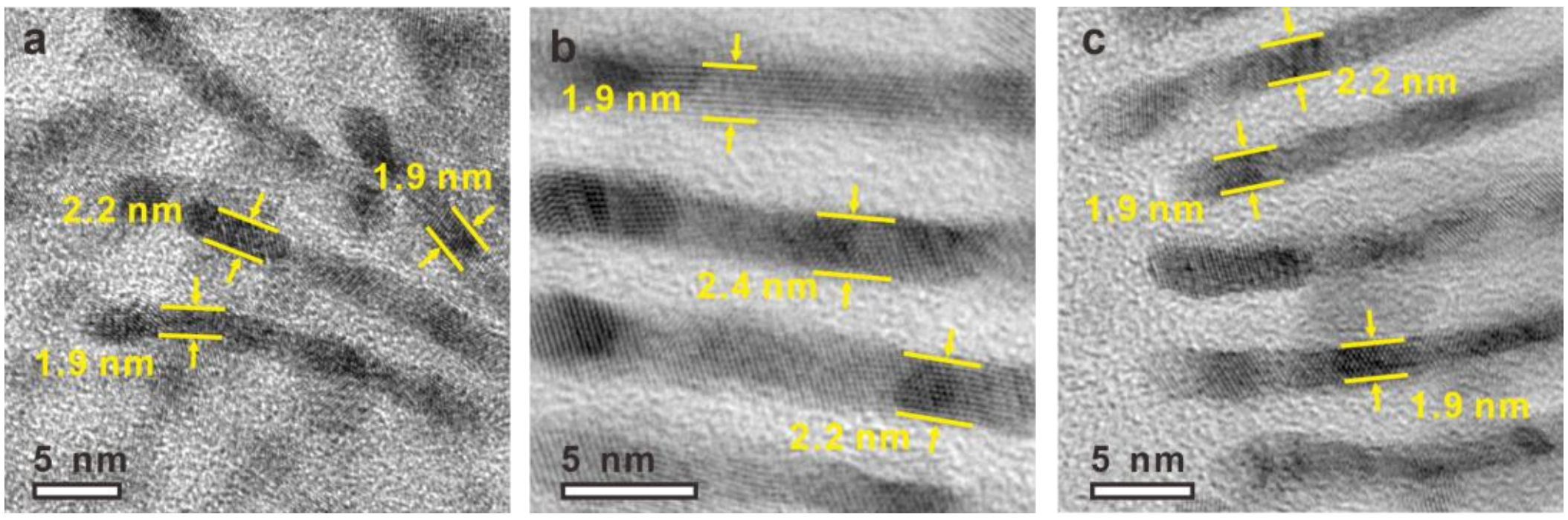

Figure S1. HRTEM images of Pd NSs viewed from the side. (a) Pd NSs_44. (b) Pd NSs_16. (c) Pd NSs_11.

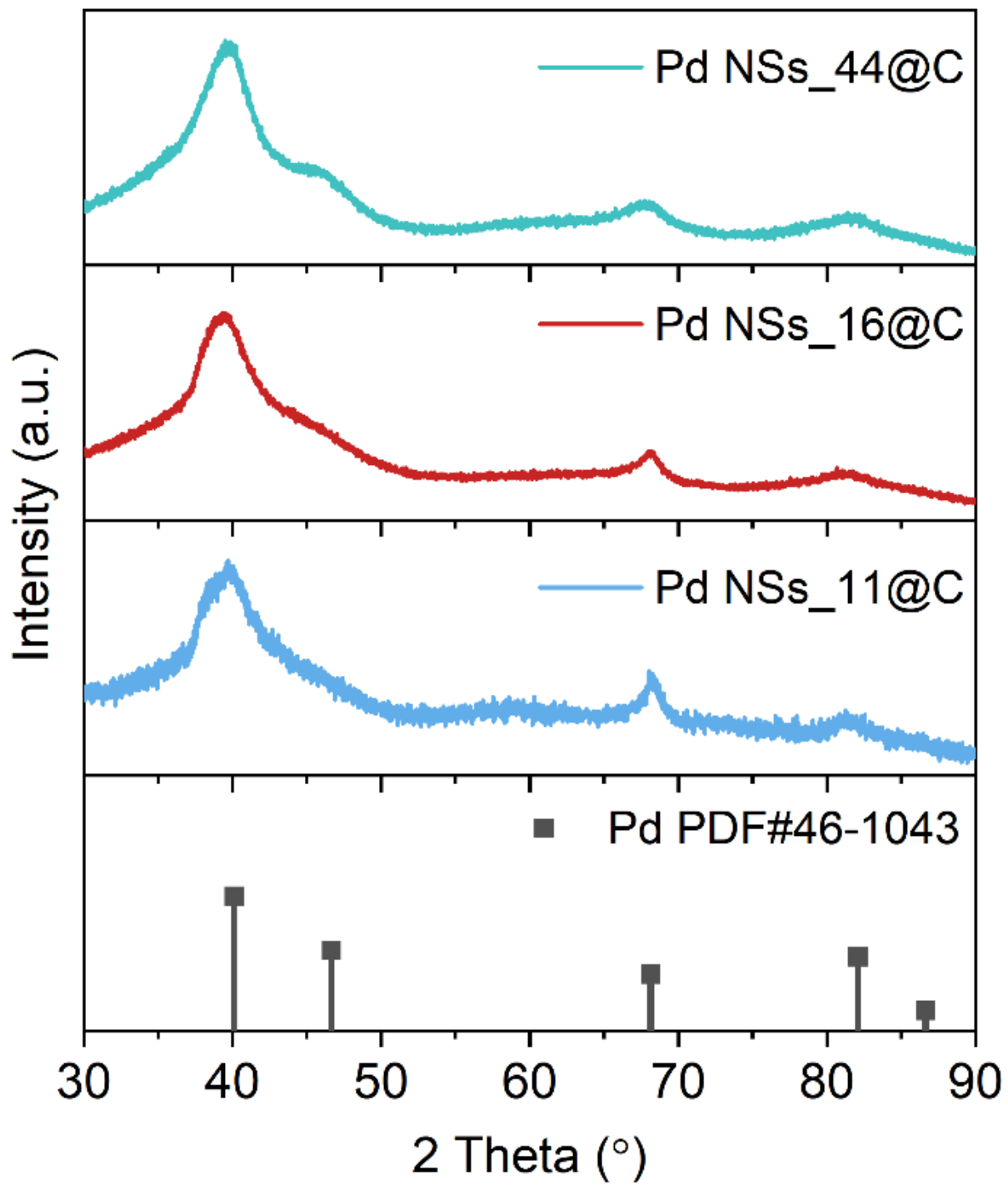

Figure S2. XRD patterns of as-prepared Pd catalysts with different sizes.

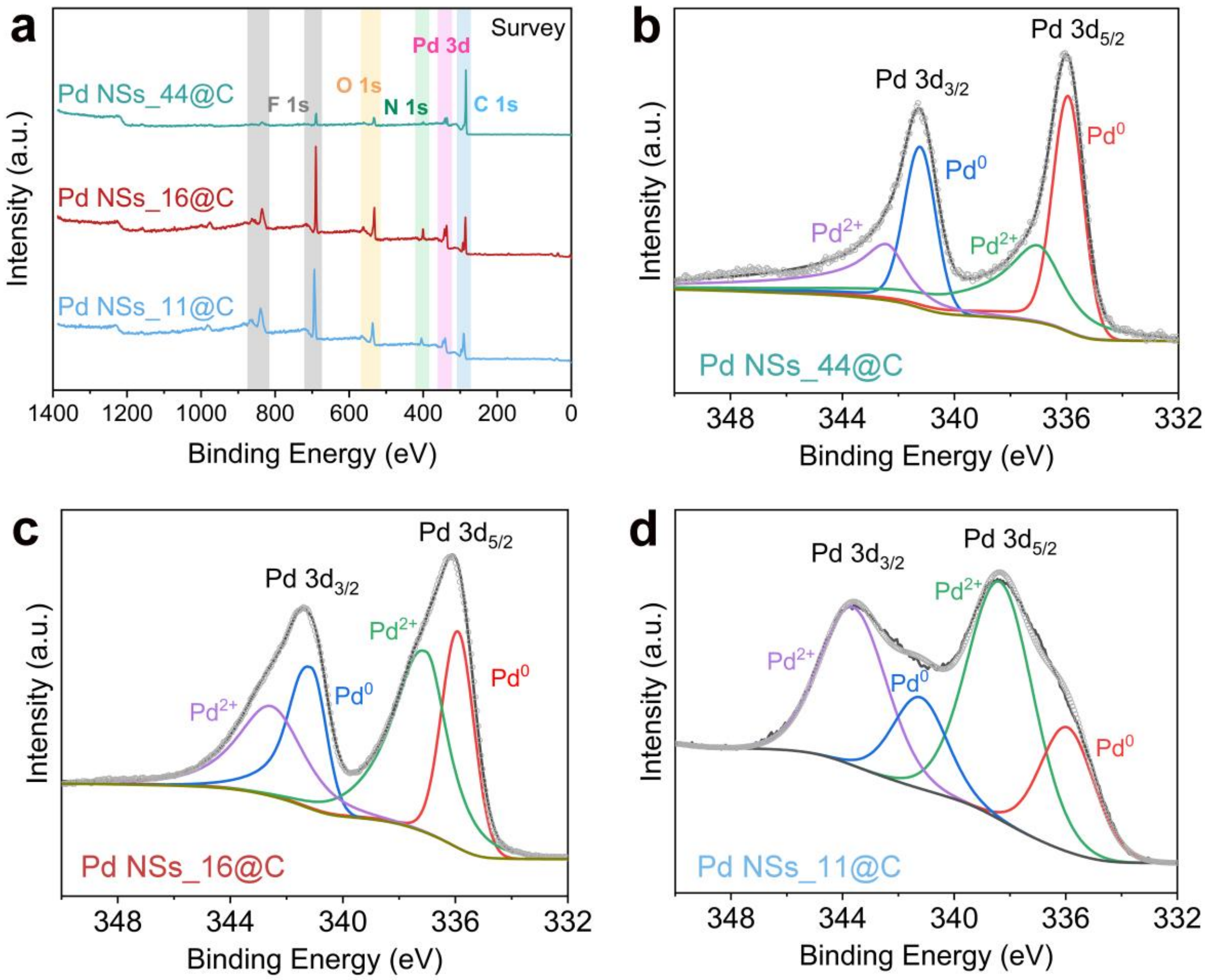

Figure S3. (a) XPS survey scan and fitted Pd 3d high-resolution core-level regions of (b) Pd NSs_44@C, (c) Pd NSs_16@C, and (d) Pd NSs_11@C.

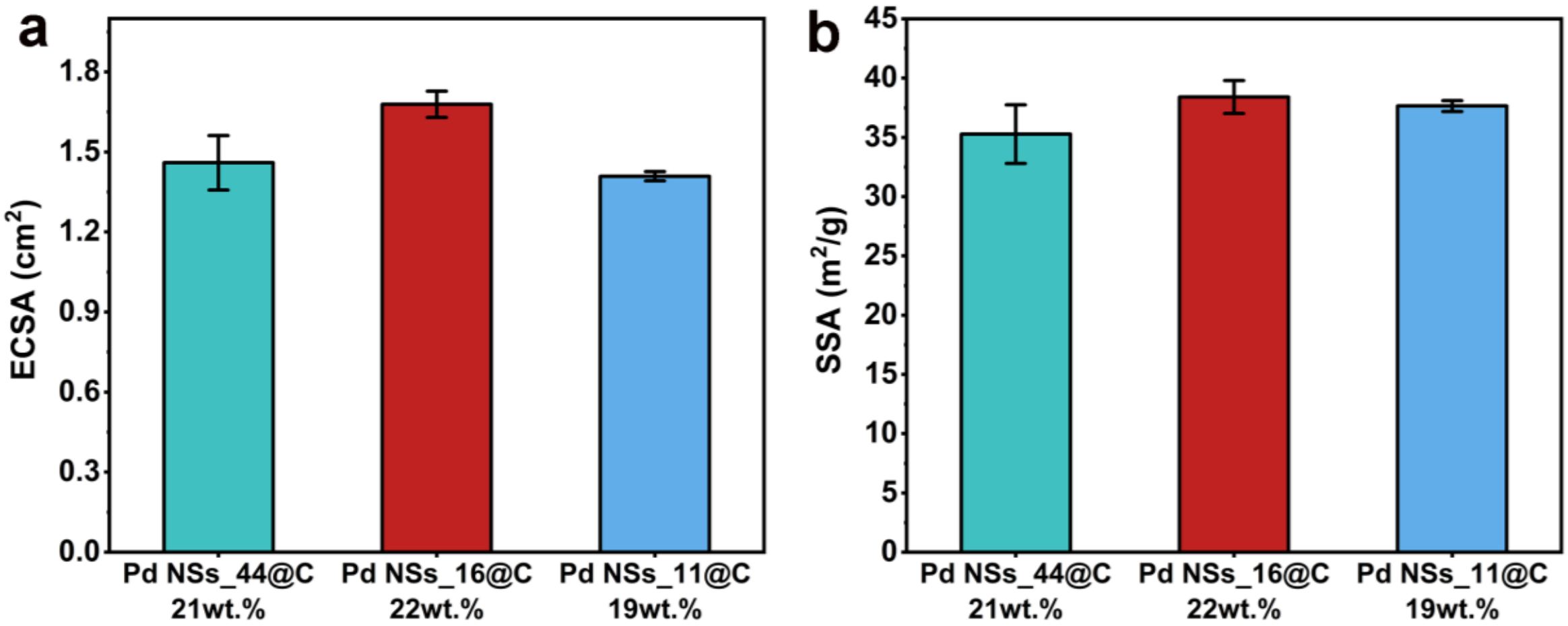

Figure S4. (a) Electrochemically active surface area (ECSA) of the three Pd NSs@C catalysts, determined from the CO-stripping measurements. (b) Specific surface area (SSA), obtained by normalizing the ECSA by the Pd mass, reflecting the accessible electrochemically active surface area per unit mass. The comparable SSA values among the catalysts indicate that lateral size modulation does not lead to substantial differences in accessible electrochemical surface area.

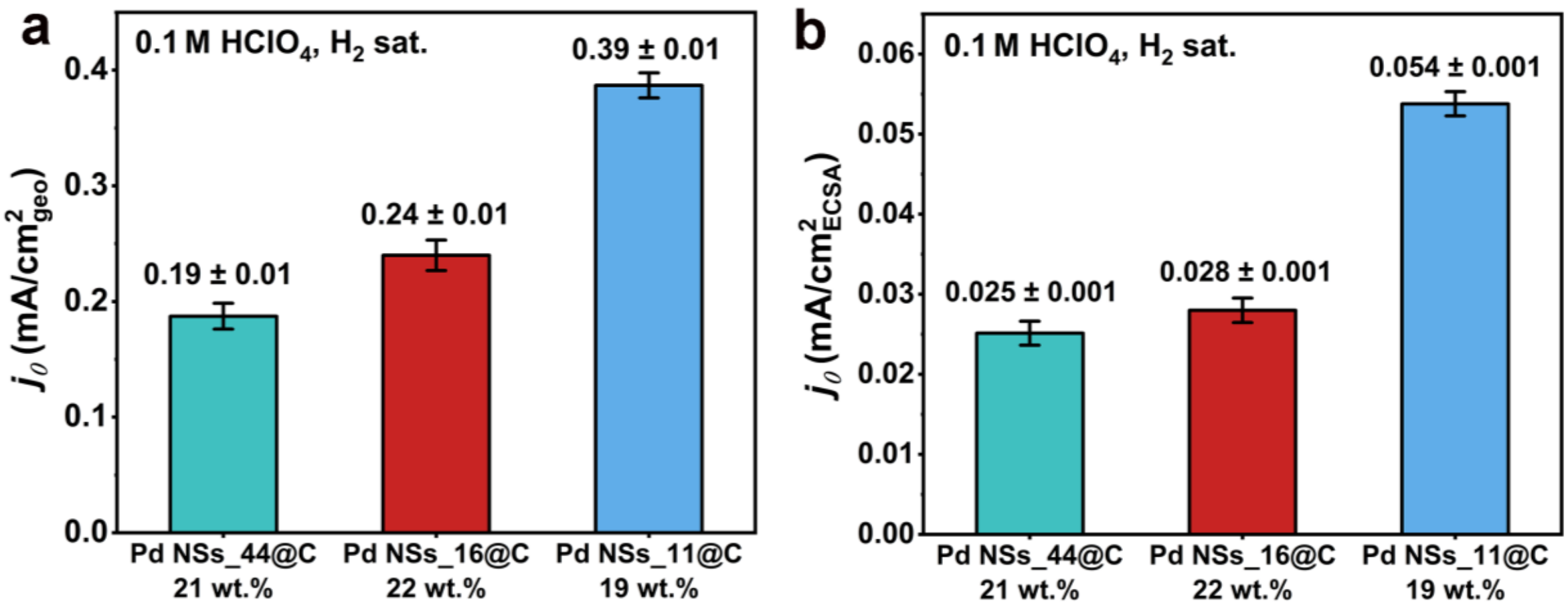

Figure S5. (a) Geometric area normalized and (b) ECSA-normalized exchange current density (j_0) of Pd NSs@C catalysts measured in H_2-saturated 0.1 M $HClO_4$. All j_0 values were extracted by Tafel extrapolation.

The Geometric area normalized j_0 values reflect the apparent HER activity influenced by both the number of accessible active sites and interfacial reaction kinetics. After the normalization by ECSA, the resulting j_0 highlights intrinsic kinetic differences of the exposed Pd surface.

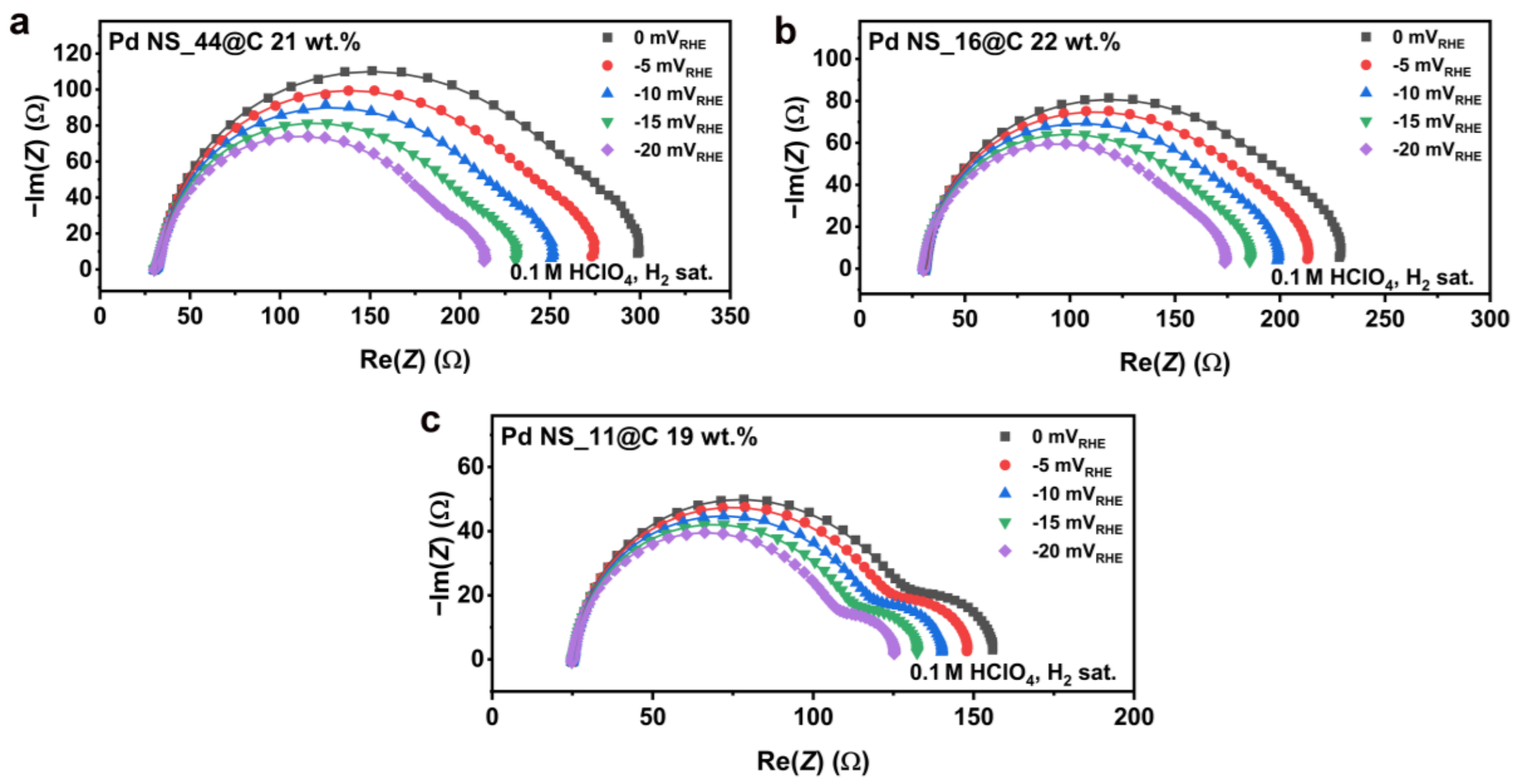

Figure S6. Nyquist EIS plots of (a) Pd NSs_44@C, (b) Pd NSs_16@C, (c) Pd NSs_11@C recorded in H_2-saturated 0.1 M $HClO_4$ at various overpotentials.

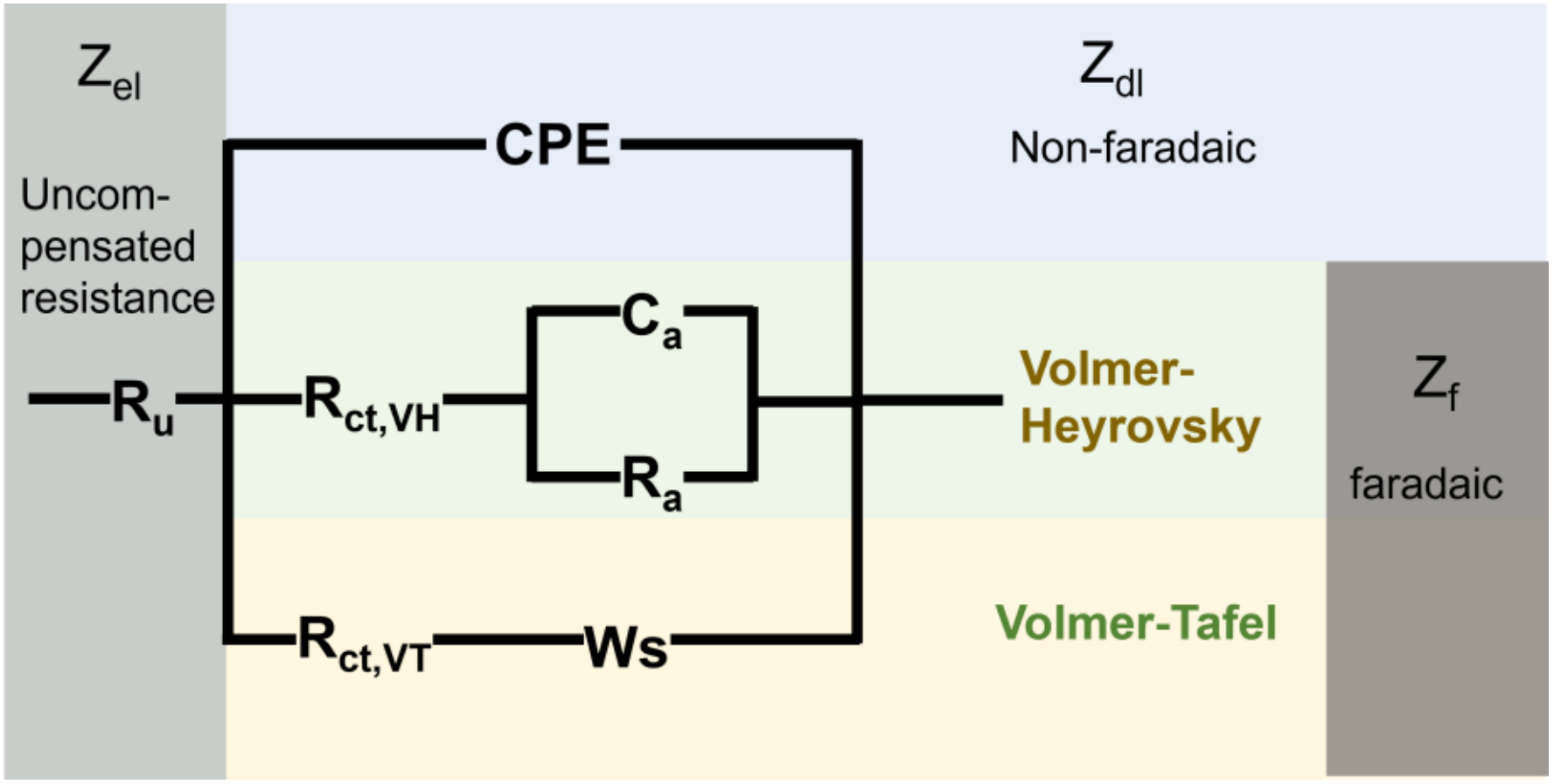

Figure S7. An equivalent electrical circuit (EEC) model used for fitting the electrochemical impedance spectra (EIS) of Pd NSs@C catalysts.

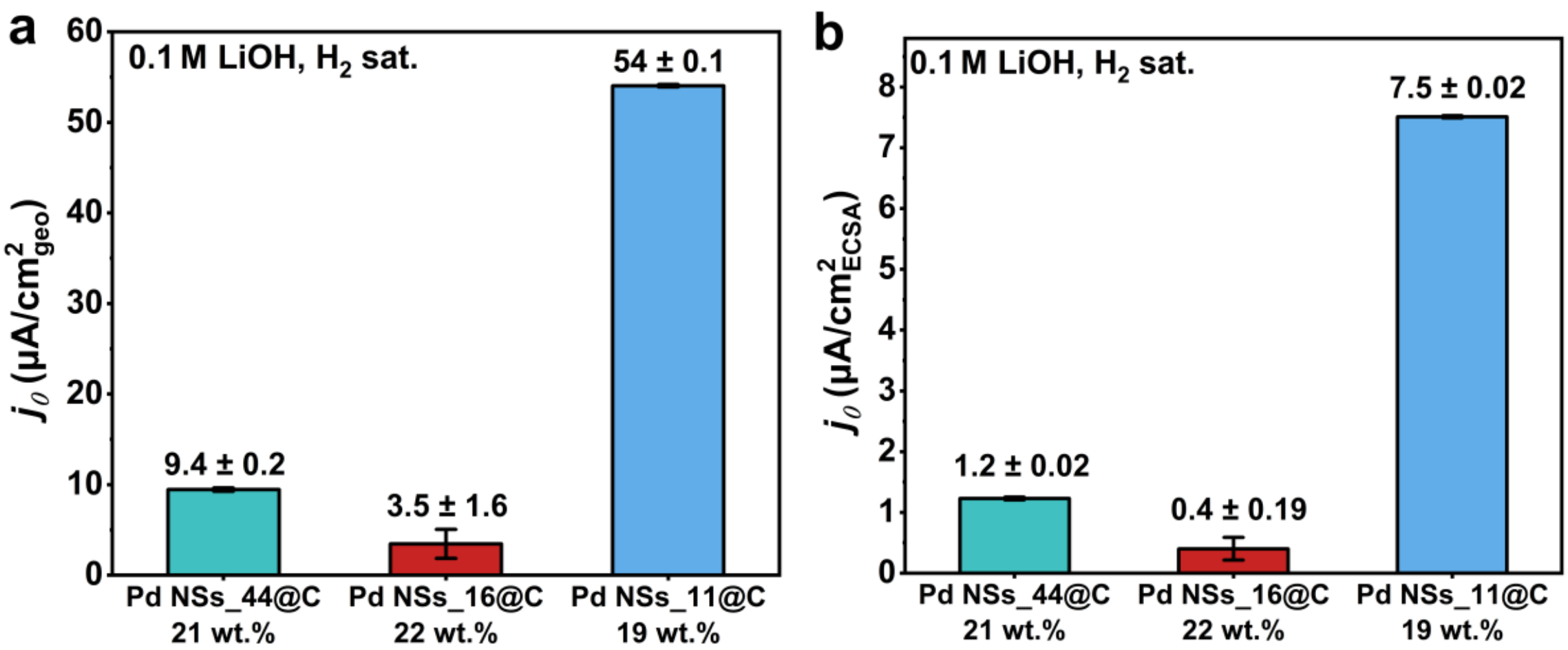

Figure S8. (a) Geometric area normalized and (b) ECSA-normalized exchange current density (j_0) of Pd NSs@C catalysts measured in H_2-saturated 0.1 M LiOH. All j_0 values were extracted by Tafel extrapolation.

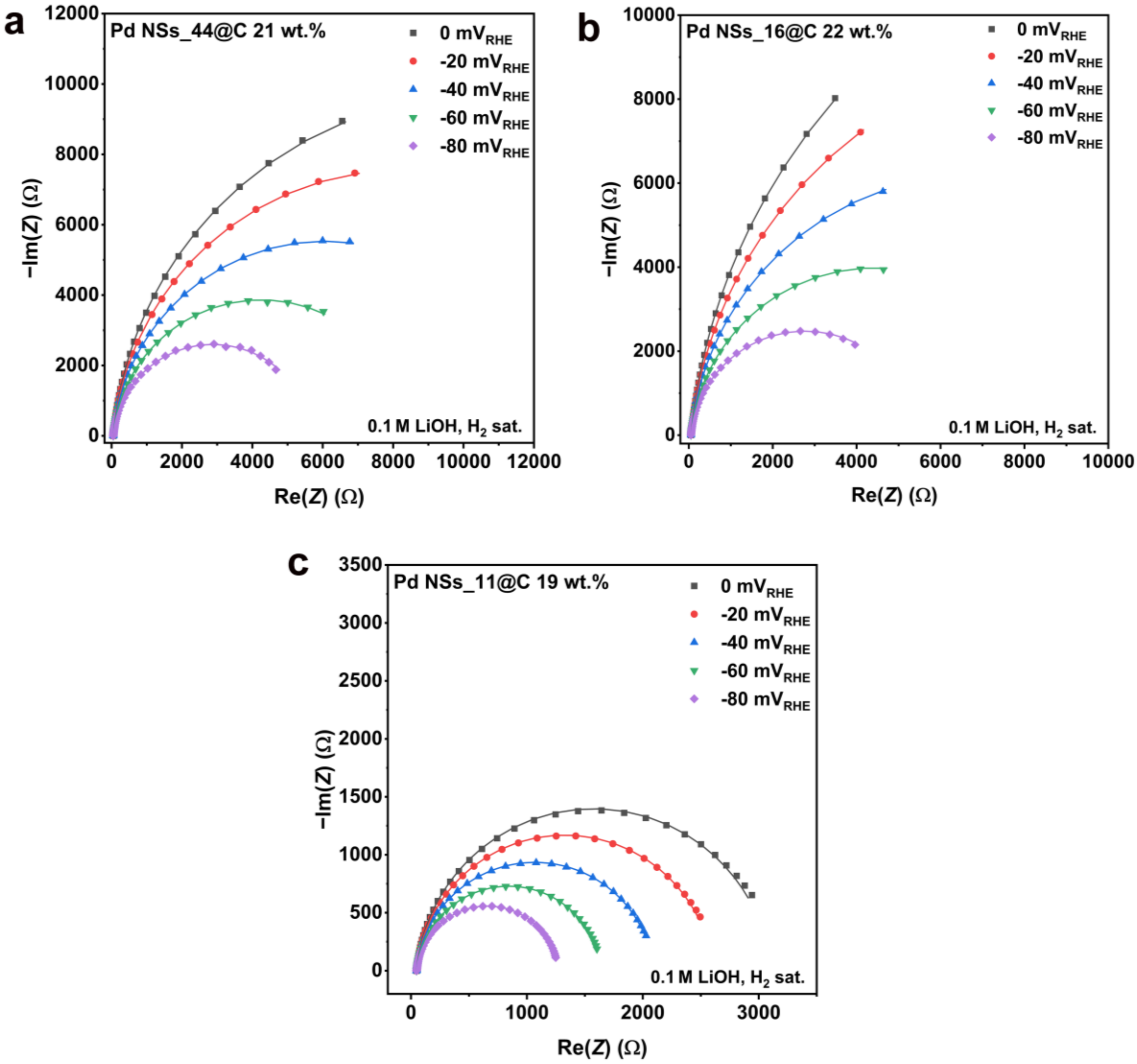

Figure S9. Nyquist EIS plots of (a) Pd NSs_44@C, (b) Pd NSs_16@C, (c) Pd NSs_11@C recorded in H_2-saturated 0.1 M LiOH at various overpotentials.

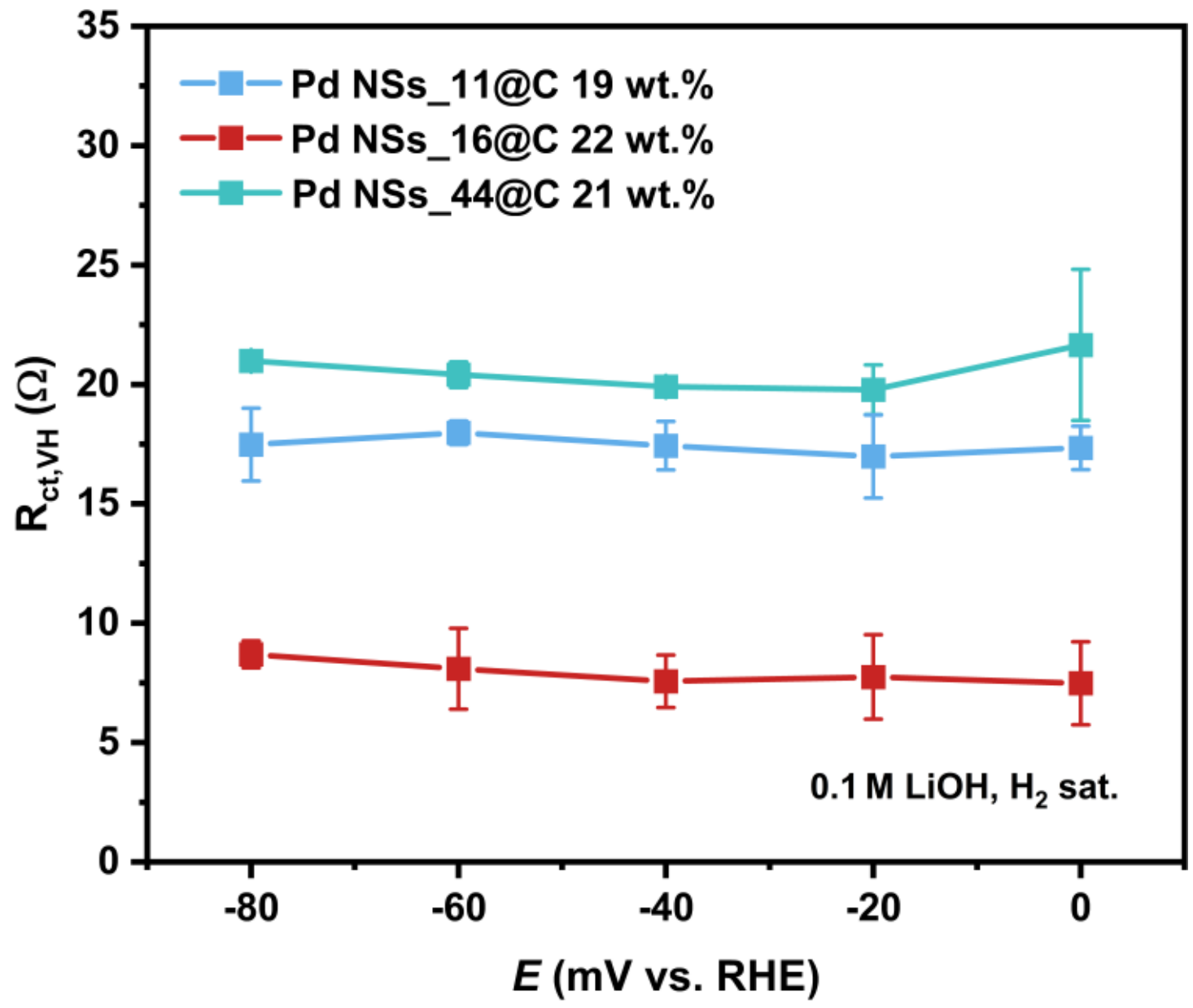

Figure S10. Charge transfer resistances extracted from the EIS fitting ($R_{ct,VH}$), measured from 0 to -80 mV vs. RHE.

Table S1. Geometric, mass, and specific HER activities of Pd NSs@C catalysts at -20 mV vs. RHE in 0.1 M $HClO_4$.

Catalysts	-GA (mA/cm^2_{geo})	-MA (mA/mg_{Pd})	-SA ($\mu A/cm^2_{ECSA}$)
Pd NSs_44@C	0.5±0.05	22.6±2.37	64.0±6.73
Pd NSs_16@C	0.6±0.01	27.9±0.46	72.1±1.19
Pd NSs_11@C	1.0±0.06	51.8±3.29	137.5±8.73

Table S2. Charge-transfer resistances of Pd NSs@C catalysts at -20 mV vs. RHE in 0.1 M $HClO_4$.

Catalysts	$R_{ct,VH}$ (Ω)	$R_{ct,VT}$ (Ω)	$R_{ct,VT}$ /$R_{ct,VH}$
Pd NSs_44@C	28.9±11.32	485.1±55.37	17.8±5.1
Pd NSs_16@C	15.4±2.10	353.5±36.33	23.3±3.3
Pd NSs_11@C	17.6±0.53	187.1±24.06	10.7±1.7

Table S3. Geometric, mass, and specific HER activities of Pd NSs@C catalysts at -80 mV vs. RHE in 0.1 M LiOH.

Catalysts	-GA (mA/cm^2_{geo})	-MA (mA/mg_{Pd})	-SA ($\mu A/cm^2_{ECSA}$)
Pd NSs_44@C	0.03±0.0003	1.6±0.02	4.4±0.04
Pd NSs_16@C	0.01±0.0062	0.6±0.28	1.4±0.72
Pd NSs_11@C	0.16±0.0038	7.3±0.17	22.3±0.52
